# Limitations of using COX proportional hazards model in cardiovascular research

**DOI:** 10.1186/s12933-024-02302-2

**Published:** 2024-06-26

**Authors:** Nan Jiang, Yongfa Wu, Chengjia Li

**Affiliations:** 1 Geriatric Psychiatry, Second People’s Hospital of Zhoushan, Zhoushan, 316000 China; 2 Heilongjiang University of Chinese Medicine , Harbin, 150000 China

**Keywords:** Triglyceride-glucose index, COX model, Right-censored

## Abstract

The article by Zhao et al. titled “Associations of Triglyceride-Glucose (TyG) Index with Chest Pain Incidence and Mortality among the U.S. Population” provides valuable insights into the positive correlation between the TyG index and chest pain incidence, as well as a nonlinear relationship with mortality. However, the use of the COX proportional hazards model in their analysis presents several limitations. The assumption of constant hazard ratios over time may not hold, potentially leading to biased estimates. The model’s struggle with time-dependent covariates and the possibility of residual confounding are notable concerns. Additionally, the study’s subgroup analyses might suffer from reduced statistical power, and potential interactions with other metabolic markers were not explored. Considering these limitations, future research should adopt alternative approaches, such as time-varying covariate models, to provide a more comprehensive understanding of the relationship between the TyG index and cardiovascular outcomes.

We read with great interest the recent article by Zhao et al. titled “Associations of Triglyceride-Glucose (TyG) Index with Chest Pain Incidence and Mortality among the U.S. Population” in *Cardiovascular Diabetology* [1]. The results of this study show that TyG is positively correlated with the incidence of chest pain, and this association remains significant after adjusting for multiple covariates. In the highest TyG quartile, for each unit increase in TyG, the incidence of chest pain increases by 42% (quartile 4 vs. quartile 1, odds ratio [OR] 1.42, 95% confidence interval [CI] 1.14–1.77, *P* = 0.002). The study also confirmed that there is a significant nonlinear relationship between TyG and mortality, regardless of whether the patients experienced chest pain. The study provides valuable insights, but we note several limitations in using the COX proportional hazards model [2].

The COX model assumes constant hazard ratios over time (proportional hazards assumption), which may not hold in practice as the effect of the TyG index on mortality risk could vary over the follow-up period. Violation of this assumption may lead to biased estimates and inaccurate conclusions. Additionally, the model struggles with time-dependent covariates such as body weight and blood glucose levels, which likely change over time, potentially misestimating the true effect of the TyG index [3].

Despite controlling for many known confounders, residual or unmeasured confounding remains a possibility. Factors like genetic predispositions, lifestyle choices, and environmental influences were not accounted for, affecting observed associations. Subgroup analyses may suffer from reduced statistical power due to smaller sample sizes within each subgroup, leading to unstable estimates and wider confidence intervals. The study also overlooks potential interactions between the TyG index and other metabolic markers like cholesterol levels, which might oversimplify the relationships between metabolic health and cardiovascular outcomes [4].

In addition, the study utilized data from the 2001–2012 National Health and Nutrition Examination Survey (NHANES) and linked participant records with the National Death Index public access files as of December 31, 2019, to determine participant endpoints and follow-up information. For participants who did not die during the study period, their follow-up time was recorded up to the date of their last contact, making this portion of the data right-censored [5].

In conclusion, while Zhao et al. provide important insights, considering the limitations of the COX proportional hazards model is crucial. Future research should explore alternative approaches, such as time-varying covariate models, to offer a more comprehensive understanding of these relationships.

## Data Availability

No datasets were generated or analysed during the current study.
